# A Retrospective Public Health Assessment and Management in Terms of the Social and Clinical Risk Factors of Respiratory Syncytial Virus Infection in Northern Canada

**DOI:** 10.7759/cureus.53378

**Published:** 2024-02-01

**Authors:** Sana Sharif Sheikh, Hina Sharif, Nadia Sharif

**Affiliations:** 1 Epidemiology and Public Health, University of Saskatchewan School of Public Health, Saskatoon, CAN; 2 Health Sciences, Agha Khan University Hospital, Karachi, PAK; 3 Pulmonology, Hera General Hospital, Makkah, SAU

**Keywords:** public health planning, bronchitis, nunavut, lower respiratory tract infection, rsv infection

## Abstract

Background

The predominant source of respiratory infections in Northern Canada stems from RSV, leading to potentially life-threatening lower respiratory tract infections in children below the age of 2. Typically, RSV begins to appear in November or December and persists until April or May. Synagis® (Palivizumab), a monoclonal antibody, is employed to mitigate or reduce the effects of RSV. Past research indicated a reduction in hospitalizations with the use of Synagis®.

Aim

The aim is to estimate the cost-benefit analysis by comparing the health services cost with Synagis^®^ program cost. Also evaluate the association of identified risk factors with the severity of RSV infection.

Material and methods

The dependent variable is categorized as: “Mild-Medium” cases that didn’t undergo intubation or require medical evacuation; “Severe” cases that underwent intubation, required medical evacuation, and intensive care unit facilities. We also calculate the cost of health services and Synagis^®^ of each year.

Results

It has been found that babies who exclusively breastfed and regularly took vitamin D did not develop severe forms of infection. Prenatal smoking and shared and crowded accommodations contribute to the spreading of RSV. The average cost of health services per participant was higher than that of the Synagis program.

Conclusion

They are promoting the Synagis® program during the season. Standardize the regulations prohibiting smoking around small children since they are more vulnerable to infection. Practice breastfeeding up to 24-month-old babies.

## Introduction

The first chimpanzee to show cold symptoms from a respiratory syncytial virus (RSV) infection was reported [1]. The RSV infection was discovered shortly after in Baltimore in small infants who had symptoms of severe respiratory illness [2,3]. Babies throughout the globe get RSV most often, making it the top cause of pneumonia and bronchiolitis [4]. RSV infection caused 3.4 million hospital admissions up till 2011 and around 66,000-199,000 fatalities annually throughout the world [5].

Due to different infection patterns and sickness with another well-known respiratory tract viral disease, RSV presents various challenges for epidemiologists [4]. RSV usually starts to manifest in November or December and lasts into April or May [6]. In Northern Canada, particularly in Nunavut, it is one of the leading causes of lower respiratory tract infections, which may have catastrophic outcomes such as bronchitis, bronchiolitis, and pneumonia [7].

A monoclonal antibody called Synagis® (Palivizumab) is used to counteract or lessen the impact of RSV. Previous studies showed that Synagis® decreased hospitalization by 55% for preterm babies with Broncho Pulmonary Dysplasia (BPD) [8].

Infant RSV infection is a frequent reason for medical flights in Nunavut to provide treatment. Children who need critical care must be transported to Qikiqtani General Hospital in Iqaluit or through Medevac or Schedevac to other authority hospitals in Southern Canada. Synagis® is used in young children under the age of two during the RSV season to lower the morbidity and death risk associated with RSV in babies. However, to ensure safety and boost protection against infection, each kid must get monthly vaccine doses during the entire RSV season.

According to the literature study, several variables might raise or reduce a child's chance of contracting an illness before the age of two. It has been shown in previous research that breastfeeding exclusively protects against RSV [9] and that children who are not being breastfed or are not being exclusively breastfed have a greater risk of infection [10]. Smoking has been connected to a higher risk of RSV during or before pregnancy [11,12].

Smoking during pregnancy by mothers increases the likelihood of developing a severe RSV infection. Smoking inside has been connected to a higher risk of RSV [13-15]. There was a very high risk of RSV infection in school-aged siblings [12-13]. The risk of catching RSV is higher in households with more than two people. Sharing a bedroom with another person also increases the risk of catching RSV [13].

RSV infections and hospitalization because of immature lungs are related to both preterm and low birth weight [13,16]. RSV risk is higher in preterm infants with chronic lung disease (CLD), such as asthma and bronchopulmonary dysplasia (BPD) [16,17]. Even in healthy full-term newborns, vitamin D deficiency is linked to severe RSV [18,19]. Increased risks of RSV complications are associated with congenital heart disease (CHD) [13,20-22].

The aim of the study is to determine the health services costs related to RSV infection and Synagis® program cost during the five-year data in order to estimate the cost-benefit analysis by comparing the cost of health services with Synagis® program cost in order and evaluate the association of identified risk factors with the severity of RSV infection.

## Materials and methods

Sample

For this study, we looked back at data from all babies in Nunavut towns who had a confirmed case of RSV between 2011 and 2016, specifically those between 0 and 24 months old. The data were collected across a five-year period.

Data sources

Test Details

Nasal swab collection was used to take the samples and use Rapid RSV antigen test to confirm the RSV cases.

Databases

The Health Protection Unit of the Government of Nunavut maintained the Nunavut Influenza and RSV Enhanced Surveillance Database, which included records of laboratory-confirmed cases of RSV together with demographic information. 

This study drew on data extracted from the Discharge Abstract Database (DAD), a repository of information on patient discharges from all Canadian healthcare institutions maintained by the Canadian Institute for Health Information. The data included hospitalization records, intubation markers, and associated expenses. 

The database of all medical travel covered by the Nunavut Health Insurance Program was queried to acquire information on medical travel, including cost. Data about Synagis® was sourced from the Nunavut Synagis® Database maintained by the Health Protection Unit of the Government of Nunavut. The Health Protection Unit of the Government of Nunavut covers the cost of Synagis®.

MEDITECH, Nunavut's electronic medical health record system, was queried for information on emergency department visits, including temporary health condition numbers (HCNs) and risk factors. We searched the patient's MEDITECH file for pertinent information in the following places: reports of pediatrician consultations, discharge summaries, well-baby records, and healthcare visits.

The Canadian Institution of Health Information provides the average cost of a standard hospital stay broken down by province and territory.

Variables

**Figure 1:**
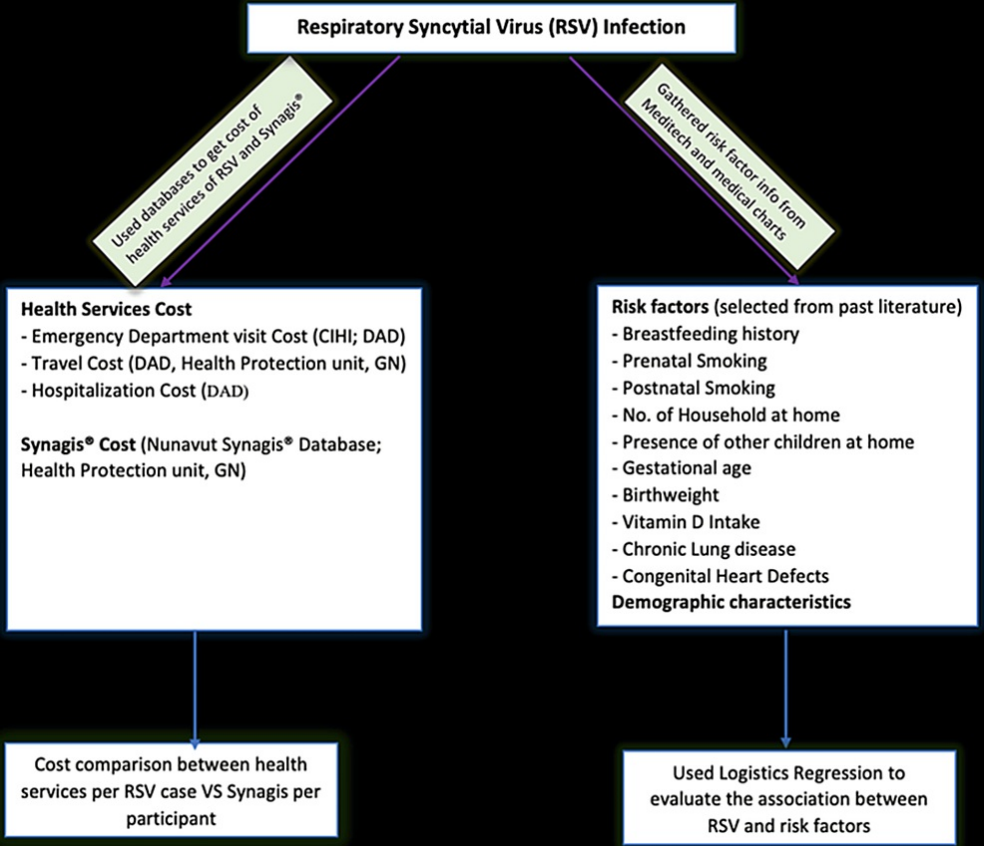
Study methodology

We used “severity of infection” as the dependent variable and divided cases into two categories: “Mild-Medium” cases were those who did not undergo intubation and did not require medical evacuation because of RSV infection; “Severe” cases were those who underwent intubation. The risk variables we considered were history of breastfeeding, exposure to prenatal and postnatal cigarette smoke, birthweight, gestational age at birth, history of vitamin D consumption, presence of other children at home, type of housing, number of households, and history of CHD, BPD, and CLD. Refer to the Supplementary table to check the inclusion criteria. The complete details of the variables and their respective definitions are explained in the Supplemental table. Refer to Figure 1 for study design and methodology.

Data Analysis

The data entered and verified in Microsoft Excel before export to SPSS version 24 (IBM Corp., Armonk, NY) for analysis, underwent rigorous scrutiny for accuracy and consistency. Data were summarized by descriptive statistics in the form of case counts and proportions.

Cost Analysis

Using data on hospitalization, medical travel, and emergency department visits broken down by respiratory season, we were able to determine the average cost of medical services. Age, season of infection, and severity will also be used to stratify the average cost of medical treatments. For each year that a patient was a part of the Synagis® program, we determined the overall cost. Regarding the cost computation, please see supplementary table.

Risk factors with the severity of RSV infection

We conducted the bivariate unadjusted analysis between the severity of infection (mild-medium vs severe) with predictor variables to screen the variables for the multivariate regression (significance level p-value less than and equals 0.20). We performed multivariate logistics regression between the outcome variable and the predictors significantly associated with bivariate analysis (significance level: p-value less than 0.05). The missing values were treated through multiple imputation methods to improve the significance level.

## Results

Descriptive statistics

Table 1 summarizes the descriptive analysis of five-year retrospective data concerning the severity of infection. Out of 461 total cases for five years, 219 cases were female, and the rest were male infants; among them, 32% were female infants, and 31% were male infants who were severely infected with RSV. The most represented age group among infected cases was from the Inuit ethnic background and the Qikiqtaaluk region; among them, 62% and 30%, respectively, were severely ill with RSV. In the study of infants, 31% were those who were severely ill and never breastfed in their early lives. 57% and 61% of infants were those with prenatal and postnatal tobacco exposure and were severely infected with RSV.

**Table 1:** Descriptive and bivariate unadjusted analysis between predictors and level of RSV infection ‡ P-value<0.01; ns: not significant

	Total n	Severity of infection		Bivariate analysis P-value < 0.20
		Severe n (%)	Mild to Medium n (%)	
Sex				‡
Female	219	147 (31.9)	72 (15.6)	
Male	242	142 (30.8)	100 (21.7)	
Age Group				ns
0-5 months	218	139 (30.2)	79 (17.1)	
6-11 months	131	80 (17.4)	51 (11.1)	
12-17 months	75	47 (10.2)	28 (6.1)	
18-23 months	37	23 (5.0)	14 (3.0)	
Ethnicity				ns
Inuit	456	286 (62.0)	170 (36.9)	
Non-Native	4	3 (0.7)	1 (0.2)	
Details not available	1	0 (0)	1 (0.2)	
Territory Regions				‡
Qikiqtaaluk	248	140 (30.4)	104 (22.5)	
Kivalliq	166	112 (24.3)	56 (12.4)	
Kitikmeot	47	37 (8.0)	12 (2.6)	
Breastfeeding^†^				‡
Exclusively	137	111 (24.1)	26 (5.6)	
Less exclusively	98	34 (7.4)	64 (13.9)	
Never breastfed	226	144 (31.2)	82 (17.8)	
Prenatal smoking^†^				‡
Yes	427	261 (56.6)	166 (36.0)	
No	34	28 (6.1)	6 (1.3)	
Postnatal smoking^†^				‡
Yes	452	280 (60.7)	172 (37.3)	
No	9	9 (2.0)	0	
No. of household^†^				‡
2-5	196	129 (28.0)	67 (14.5)	
6-10	168	93 (20.2)	75 (16.3)	
>10	97	67 (14.5)	30 (6.5)	
Other children at home^†^				‡
Yes	367	221 (47.9)	146 (31.7)	
No	94	68 (14.8)	26 (5.6)	
Gestational age^†^				
<36 weeks	300	200 (43.4)	100 (21.7)	‡
>36 weeks	161	89 (19.3)	72 (15.6)	
Birth weight^†^				‡
<2500 grams	288	189 (41.0)	99 (21.5)	
>2500 grams	173	100 (21.7)	73 (15.8)	
Vitamin D intake^†^				‡
Never	268	169 (36.7)	99 (21.5)	
Sometimes	119	81 (17.6)	38 (8.2)	
Always	74	39 (8.5)	35 (7.6)	
Type of accommodation^†^				‡
Shared	411	253 (54.9)	158 (34.3)	
Private	50	36 (7.8)	14 (3.0)	
Chronic lung disease^†^				
Yes	200	115 (24.9)	85 (18.4)	
No	261	174 (37.7)	87 (18.9)	
Congenital heart defects^†^				‡
Yes	250	129 (28.0)	121 (26.2)	
No	211	160 (34.7)	51 (11.1)	
‡ P-value<0.01; ns: not significant				

There were 20% of cases severely ill with RSV lived with six to 10 people at home, and among them, 48% of severe cases lived with other children at home, and 55% of severely ill cases lived in shared accommodation. In the study infants, 43% of infants with severe RSV were born premature (gestational age < 36 weeks), and 41% were those with lower birthweight (<2,500 grams). 37% of severe RSV cases never intake vitamin D, 25% of severe RSV cases had a condition of CLD, and 28% of severely infected RSV cases had a condition of CHD.

Multivariate analysis between RSV risk factors with severity of infection

Table 1 also shows the unadjusted bivariate analysis between risk factors and the level of RSV infection. All the predictor variables other than age and ethnicity were significantly associated in unadjusted bivariate analysis. So, we excluded the age and ethnicity in the adjusted multivariate regression model.

Table 2 represents the multivariate logistics regression between the severity level of RSV infection (outcome variables) and risk factors (predictor variables) gathered from past literature. The odds of developing RSV among infants who never breastfed was 2.282 times the odds of developing RSV who exclusively breastfed (p-value<0.001; 95% CI: 1.254-4.154). The odds of developing RSV among children whose mothers had prenatal use of tobacco smoking were 4.45 times the odds of those who were not exposed to tobacco smoking (P-value<0.05; 95% (Cl: 1.511-13.134). The odds of developing RSV infection among infants who live with other children at home were 2.65 times the odds of those who did not live with other children at home (P-value<0.05; 95% (Cl: 1.404-5.007). The odds of developing RSV among infants who live with more than ten people at home were 3.168 times the odds of those who live with 2-5 people at home (P-value<0.05; 95% (Cl: 1.651-6.081). The odds of developing RSV infection among young infants who never take vitamin D were 0.371 times the odds of developing among those who always take vitamin D (P-value<0.05; 95% (Cl: 0.177-0.774). The odds of developing RSV infection among children with CHD condition were 3.66 times the odds of developing RSV who did not have any CHD condition (P-value<0.05, 95% Cl: 2.208-6.085). The odds of developing RSV among children who lived in shared accommodation were 2.81 times the odds of developing among those who lived in private accommodation (P-value<0.05; 95% (Cl: 1.162-6.780).

**Table 2:** Multivariate adjusted analysis between predictors and level of RSV infection ¥: P-value < 0.05; ‡: P-value<0.001; €: Confounding effect; ns: not significant; Ref: Reference category; AOR: Adjusted odd ratio 95% CI: 95% Confidence Interval

RSV risk factors	AOR	95% CI	P-value
Breastfeeding			‡
Never breastfed	2.282	1.254-4.154	
Less exclusively	0.132	0.069-0.251	
Exclusively	Ref	-	
Prenatal smoking			¥
Yes	4.454	1.511-13.134	
No	Ref	-	
Postnatal smoking^€^			ns
Yes	1.198	-	
No	Ref	-	
No. of household			¥
>10	3.168	1.651-6.081	
6-10	1.814	1.062-3.100	
2-5	Ref	-	
Other children at home			¥
Yes	2.651	1.404-5.007	
No	Ref	-	
Vitamin D intake			¥
Never	0.371	0.177-0.774	
Sometimes	0.900	0.514-1.577	
Always	Ref	-	
Type of accommodation			¥
Shared	2.806	1.162-6.780	
Private	Ref	-	
Congenital heart defects			‡
Yes	3.665	2.208-6.085	
No	Ref	-	
Chronic lung disease^†^			ns
Yes	-	-	
No	Ref	-	
Gestational age^†^			ns
<36 weeks	-	-	
>36 weeks	Ref	-	
Birth weight^†^			ns
<2500 grams	-	-	
>2500 grams	Ref	-	
¥: P-value < 0.05; ‡: P-value<0.001; ns: not significant; €: Confounding effect; Ref: Reference category AOR: Adjusted odd ratio; 95% CI: 95% Confidence Interval			

Our data indicated that there was no association between birth weight, CLD, post-natal smoking, and developing RSV infection among study children. However, postnatal smoking has a potential confounding effect on predictor variables; therefore, we have included it in the model.

Cost analysis

Health Services Cost

A total of around $5,625,649 was spent on all medical treatments over the five-year period of RSV infection, with an average cost of about $12,203 per case, according to the data. With regard to the season of infection, Table 3 displays the overall expenditures of medical services together with an average cost per case. Additionally, Table 3 displays the breakdown of medical care expenditures by infection year, including ER, travel, and hospitalization expenses. Emergency room expenses were over $235,260 higher in the 2011-12 season of infection, travel expenses were around $638,201 higher in the 2012-13 season of infection, and hospitalization expenses were around $783,026 higher in the 2013-14 season.

**Table 3:** Cost analysis with respect to Year of Infection and Health Services All the cost were in Canadian Dollar

Year of Infection	Total Medical Services cost	Average medical services cost per case (n)	ER Cost (% of total cost)	Travel Cost (% of total cost)	Hospitalization Cost (% of total cost)
2011-2012	$984,570	$ 8,070 (122)	22%	36%	42%
2012-2013	$1,409,723	$ 10,928 (129)	5%	45%	50%
2013-2014	$1,367,104	$ 12,542 (109)	3%	41%	56%
2014-2015	$813,314	$ 16,651 (49)	10%	46%	44%
2015-2016	$887,627	$ 15,641 (52)	4%	37%	59%
Total Cost	$5,462,339	$ 11,849 (461)	$436,987 (8%)	2,239,559 (41%)	2,785,792 (51%)

Synagis® Program-Related Cost

There was a total of 376 Synagis® participants in five years' data (2011-2016), more of the candidates enrolled during the year 2012/13, i.e., 94 candidates (25%), whereas 80 candidates (21%) and 77 candidates (20%) during the year 2013-14 and 2014-15 respectively. 

Cost-Benefit Analysis by Comparing Health Services and Synagis® Program Cost

Over the five years that RSV was common, Table 4 breaks down the overall cost of medical services associated with the virus as well as the cost of Synagis®. On average, each participant spent roughly $7,414 on Synagis® and around $12,203 on RSV.

**Table 4:** Comparison of Synagis® & Medical Services due to RSV by Year of Infection All the cost were in Canadian Dollar

Year	Synagis® participants	Total Synagis® Cost	RSV cases	Cost of Medical Services & Travel
2011-12	61	$516,646	122	$984,570
2012-13	94	$689,070	129	$1,409,723
2013-14	80	$514,544	109	$1,367,104
2014-15	77	$563,441	49	$813,314
2015-16	64	$504,013	52	$887,627
Total	376	$2,787,713	461	$5,462,339

## Discussion

Through the descriptive test, it was discovered that there was no significant difference in the number of RSV cases among males and females. Instead, parents of infants between the age group of zero to five months had a higher likelihood of having their children tested and expected to visit a doctor. However, this is also possible because infants under six months have lower infection resistance than children in other age groups. In addition, Qikiqtaaluk is a larger region in the Nunavut territory than the other two regions, so the proportion of cases there was higher than in Kivalliq and Kitikmeot, as expected due to the higher resident populations. Additionally, Qikiqtani General Hospital, located within this region, may cause more RSV cases in Iqaluit. Similarly, Inuit make up 85% of Nunavut's population, which may explain why there are more instances (456 cases) among Inuit people than among people of other ethnicities.

An increased number of cases (129 in 2012/13) is likely responsible for the greater cost compared to other infection seasons, according to the data. Seasons 2015/16 and 2014/15 had higher average costs per case ($17,587 and $16,122, respectively). A larger percentage of patients handled outside of the area, as well as increased travel and hospitalization expenditures, are the reasons for this.

The examination of this study's data revealed that individuals with a history of nursing had significant outcomes; these cases had higher instances of severe levels of infection than those who were exclusively breastfeeding. Another notable effect of prenatal smoking is that moms of young children who smoked before or during pregnancy had more RSV infections than mothers of children who never smoked. Additionally, a weak correlation and confounding impact with RSV were reported for postnatal smoking. The number of persons living in the home also had a significant impact on the prevalence of RSV; these instances were more prevalent among households with two to five occupants. According to earlier research, if there were more school-going children present with young children or newborns, the chance of RSV would be increased. Other kids who lived with the sick kids at home also showed noteworthy associations with the illness.

Limitations of the study

The instances examined in this research involved RSV cases confirmed in the laboratory, which may lead to a potential underestimation of the true number of RSV cases. Not all individuals in the Kivalliq and Kitikmeot regions were swabbed for RSV due to the absence of specific facilities. This could potentially result in an underestimation of the true number of RSV cases in those two regions or an overestimation of the cases in the Qikiqtaaluk region. The Qikiqtani General Hospital, located in the Qikiqtaaluk region, might potentially record a higher number of hospitalized cases from the Qikiqtaaluk region, including a greater number of medium-severity cases. This is because individuals with mild RSV symptoms may also be hospitalized, contributing to the overall count.

## Conclusions

The impact of RSV requires a comprehensive approach that emphasizes the promotion of exclusively breastfeeding, the avoidance of prenatal and postnatal smoking, and the recognition of the benefits of Synagis immunization. Advocating for these measures not only can enhance the overall health and well-being of infants, but also contribute to substantial financial savings and a reduction in the burden of RSV-related diseases to the Government of Nunavut, Canada. Embracing these strategies collectively serves as a crucial step toward safeguarding the vulnerable population and fostering a healthier future for Northern Canadian communities in the Territories. Through a cost comparison, it is evident that RSV infection imposes a significant financial burden on the Government of Nunavut. Implementing the Synagis Universal program would alleviate this cost burden for the government.

Recommendations

Compared estimated cost of the universal Synagis® program, including the medical cost for the estimated no. of cases assuming effectiveness is similar in the general population. Estimated cost for universal Synagis® program = % of cases among Synagis participants (mild, medium, severe) x total population 0-23 months x average cost incurred per case (mild, medium, severe).

Place greater emphasis on minimizing the risk factors associated with RSV by collecting additional data that can comprehensively assess the overall risk scenario. In this context, formulate policies aimed at reducing smoking indoors or in the presence of young children, who are particularly vulnerable to infections.

Persist in endeavors to tackle risk factors; initial associations with risk factors indicate a heightened prevalence of tobacco smoke exposure among RSV cases and milder infections among cases exclusively breastfed for a minimum of two months.

Future activities of this program

For future reference, it is advisable to collect all risk factors beyond those available in the Meditech database, including information presented in paper charts, etc. Additionally, explore the associations between these risk factors and RSV infection. It is also recommended to investigate how these risk factors may impact the severity of infection by comparing hospitalization (with or without intubation) and non-hospitalized patients on a larger scale.

## Appendices

**Table 5:** Inclusion criteria for clinical utilization variables

Variable	Source	Criteria	Inclusion Codes in Data Set
Hospitalization	DAD	HCN of record matched patient’s HCN or mother's HCN; OR Record’s date of birth matches patient’s date of birth and HCN on record registered to individual with the same last name as patient. Date admitted in record occurred between symptom onset date and 14 days after the positive RSV swab collection date; OR Symptom onset date or date of swab collection date occurred during the hospitalization (between admitted and discharge dates). At least one inclusion code (right) recorded in record’s diagnostic codes	J00.X (Acute naso-pharyngitis); J01.X (Acute sinusitis); J02.X (Acute pharyngitis); J03.X (Acute tonsillitis); J04.X (Acute laryngitis and tracheitis); J05.X (Acute obstructive laryngitis & epiglottitis; J06.X (Acute URTIs of multiple and unspecified sites); J12.8 (Other viral pneumonia); J12.9 (Viral pneumonia. Unspecified); J15.9 (Unspecified pneumonia); J16.8 (Pneumonia due to other specified infectious organisms); J18.X (Pneumonia, unspecified organism); J20.8 (Acute bronchitis due to other specified organisms); J20.9 (Acute bronchitis, unspecified); J21.X (Acute bronchiolitis); J22.X (Unspecified acute LRTI)
Intubation	DAD	At least one procedure code (right) recorded in hospital record for patient that met inclusion criteria (above).	1.GZ 31.CA-ND, 1.GZ 31.CA-EP,1.GZ 31.CA-PK (Endotracheal Intubation); 1.GZ 31.CR-ND(Intubation through tracheostomy)
Emergency Department Visits	MEDITECH	Visit to Qikiqtani General Hospital Emergency Department noted in patient’s MEDITECH file	Visit type: DEP ER Location: IQ.ER
Medical Travel	Medical travel database	HCN of record matched patient’s HCN or mother's HCN; OR Record’s date of birth matches patient’s date of birth and HCN on record registered to individual with the same last name as patient. Travel dates occurred between symptom onset date and 14 days after the positive RSV swab collection date; OR Symptom onset date or date of swab collection date occurred between travel dates.	

**Table 6:** Variables and descriptions

Name	Valid values	Description
Patient’s Demographic Information		
HCN	Unique numeric identifier, nine digits in length	Patient’s unique lifetime identifier for Nunavut Health Insurance Registry. If unknown, unique dummy HCN assigned.
Last Name	Name up to 25 characters in length	Patient’s family name of patient linked to Health Insurance Registry file. Used to help identify clinical utilization records related to patient but recorded under caregiver’s HCN.
First Name	Name up to 25 characters in length	Patient’s given name of patient linked to Health Insurance Registry file.
Sex	Male, Female, Unknown	Biological sex of patient
Date of Birth	DD-MM-YYYY	The calendar day, month and year in which patient was born. Used to calculate age.
Age	0-5 month, 6-11 month, 12-17 months,18-23 months	Patient’s age in months on date of swab collection, calculated based on date of birth
Community	Community name (25 valid values)	Patients, or primary caregiver’s, community of residence at time of infection.
Region	Qikiqtaaluk, Kivalliq, Kitikmeot	Patients, or primary caregiver’s, region of residence at time of infection, based on community.
Ethnicity	Inuit, Non-Native, Non-Registered Metis, Registered Metis, Dene, Out of Territory	Identified using last digit of patient’s valid HCN
Caregiver’s HCN	Unique numeric identifier, nine digits in length	Patient’s caregiver’s unique lifetime identifier for Nunavut Health Insurance Registry, used to identify clinical utilization records for patient prior to being assigned own HCN
RSV Infection Information		
Symptom’s Onset Date	DD-MM-YYYY	Symptoms onset date for RSV infection, prior to positive swab collection. If not available, date four days prior to swab collection date was used as proxy
Swab Collection Date	DD-MM-YYY	The calendar day, month and year in which laboratory-confirmed RSV swab collected. Used to calculate age and year of infection.
Year of RSV Infection	(2011/12), (2012/13), (2013/14), (2014/15), (2015/16)	Respiratory season during which patient’s RSV infection took place. Calculated based on swab date.
Severity of Infection	Mild, Moderate, Severe	Proxy based on hospitalization and intubation records (see below). Mild – no hospitalization Moderate – hospitalization due to RSV, no intubation Severe – hospitalized and intubated due to RSV infection
Clinical Utilization		
Hospitalized	Yes or No	Indicator for whether patient was admitted to a hospital for care related to laboratory-confirmed RSV infection. See inclusion criteria below for further information.
Length of Stay	Number	Number of day’s patient was hospitalized for as indicated in hospital record. Used to calculate estimated hospitalization cost if no hospital cost included in record.
Province/Territory of Hospitalization	Province/Territory name	Province or Territory in which patient was hospitalized, as indicated in hospital record. Used to calculate estimated hospitalization cost if no hospital cost included in record.
Resource Intensity Weighting	Number	A comparative value listed in hospital record that refers to the estimated resource utilization of an average patient during hospitalization, as defined by the Canadian Institution of Health Information. Used to calculate estimated hospitalization cost if no hospital cost included in record.
Medical Travel	Yes or No	Indicator for whether medical travel dataset indicates travel for patient related to laboratory-confirmed RSV infection. See inclusion criteria below for further information.
Emergency Department Visit	Yes or No	Indicator for whether patient was seen in the Qikiqtani General Hospital Emergency for care related to laboratory-confirmed RSV infection. See inclusion criteria below for further information.
Intubated	Yes or No	Indicator for whether patient was intubated during hospital stay based on hospital records. See inclusion criteria below for further information.
Cost of Hospitalization	Numeric	Cost of patient’s hospital admissions related to RSV infection as listed in DAD or, if no cost included in record, estimated as per calculations outlined in cost table (below). Cost was rounded off to nearest dollar.
Cost of Medical Travel	Numeric	Cost of patient’s medical travel, including accompanying persons, related to RSV infection as listed in the medical travel database. Cost was rounded off to nearest dollar.
Estimated Cost of Emergency Department Visit	Numeric	Estimated cost of patient’s Emergency Department visit related to RSV. Calculations detailed in cost table (below). Cost were rounded off to nearest dollar.
Average Cost of Standard Hospital Stay in Province/Territory	Numeric	Used to calculate estimated hospitalization cost if no hospital cost included in record.
Synagis® History		
Synagis®	Yes or No	Indicator whether patient was enrolled in Nunavut Synagis ® Program during the respiratory season in which RSV infection occurred and received at least one dose prior to swab collection date.
Date of last dose	DD-MM-YYYY	The calendar day, month and year in which the last Synagis ® dose was administered to patient immediately prior to the swab collection date.
Up-to-Date dose	Yes, on time (≤ 28 days) Yes, not on time (>28days) No, on time (≤ 28 days) No, not on time (>28days)	Indicator of whether patient was up-to-date with Synagis® vaccination at time of RSV infection. Determined by the number of days between date of last dose and date of swab collection.
Cost of Synagis®	Numeric	Total cost of Synagis® doses administered to patient during the respiratory season in which RSV infection occurred. Further information on cost calculation included in cost table (below).
Child’s Risk Factors For severe RSV/Clinical		
Breastfeeding	Exclusively/ Not Exclusively / Never / Less than 2 months of age	For infants, older than 2 months, indicator of breastfeeding history of patient as reported by caregiver. From the birth, up to the time of infection Exclusively: only breastmilk, no formula fed to patient for more than two months Not exclusively: breastmilk fed to patient in addition to formula and/or other milk products, or exclusive breastfed for less than two months. Never: No breastmilk fed to child, only formula and/or other milk products
Prenatal Exposure to Tobacco	Yes or No	Indicator of whether biological mother reported smoking tobacco while pregnant with patient.
Postnatal Exposure to Tobacco Inside the Home	Yes or No	Indicator of whether patient lives with individuals who smoke tobacco inside of the accommodation, reported at any time prior to swab collection date.
No. of Household	2-5, 6-9, ≥10	Number of people who typically reside at patient’s primary residence as reported by caregiver.
Presence of Older Children in the Home	Yes or No	Whether older children are regularly present in the patient’s primary residence.
Gestational Age at Birth	<36 weeks or >36	Patient’s gestational age at birth in completed weeks.
Birth weight	<2500g or >2500g	Patient’s weight at birth in grams.
Vitamin D Supplements	Never, Ever, Sometimes, Always	Indicator whether patient was prescribed but given Vitamin D supplements reported at any time prior to swab collection date. Never: never given Ever: given at least once Sometimes: given at least monthly Always: given at least five times a week
Type of Accommodation	Private or Shared	Private: only patient's immediate family shared the primary accommodation; Shared: patient's primary accommodation shared by individuals other than patient’s immediate family.
Chronic Lung Disease	Yes or No	Indicator of whether patient was diagnosed by a physician with a chronic lung disease at any point prior to RSV infection.
Congenital Heart Disease	Yes or No	Indicator of whether patient was diagnosed by a physician with congenital heart disease at any point prior to RSV infection.

**Table 7:** Cost calculation

Variable	Source	Calculation
Cost of Hospitalization	DAD	Cost of patient’s hospital admissions related to RSV infection as listed in DAD or, if no cost included in record, estimated with following formula: Length of Stay X Resource Intensity Weight (RIW) X Average Cost of Standard Hospital Stay in Province/Territory
Estimated Cost of Emergency Department Visit	CIHI – average cost of stay DAD - RIW	Estimated cost of an Emergency Department Visit was calculated by multiplying the average cost of a standard one-day hospital stay in Nunavut by the Resource Intensity Weight (RIW) of a one-day hospital stay with only one procedure for a child under 2 years old age. This cost was calculated for each respiratory season as the average cost of a standard hospital stay changes from year to year. Average Cost of Standard Hospital Stay in Nunavut X Resource Intensity Weight (patient under 2 years of age, one procedure during stay)
Cost of Synagis®	Nunavut Synagis® Database – number of doses, dose size Cost of Synagis® is taken from Health Protection unit, GN.	Cost of Synagis® per patient was calculated by multiplying the number of vials used for the patient by the vial price (2015/16 price list). Number of doses and size of doses were listed in Nunavut Synagis® Database for 2011/12, 2012/13 and 2013/14. When no dose sizes were listed for a patient, cost was estimated using the average cost for patients who received the same number of doses in a respiratory season. Estimated Cost of Synagis® is calculated by the average Cost of Synagis® in a year divided by the No. of participants in the same year.

## References

[1] Morris JA, Blount Jr RE, Savage RE (1956). Recovery of cytopathogenic agent from chimpanzees with goryza. Proc Soc Experiment Biol Med.

[2] Chanock R, Roizman B (1957). Recovery from infants with respiratory illness of a virus related to chimpanzee coryza agent (CCA). I. Isolation, properties and characterization. Am J Hygiene.

[3] Chanock RM, Parrott RH, Johnson KM, Mufson MA, Knight V (1963). Biology and ecology of two major lower respiratory tract pathogens-RS virus and Eaton PPLO. Perspectives Virol.

[4] Mclntosh K Respiratory syncytial virus. Viral Infect Humans.

[5] Nair H, Nokes DJ, Gessner BD (2010). Global burden of acute lower respiratory infections due to respiratory syncytial virus in young children: a systematic review and meta-analysis. Lancet.

[6] McCarthy CA, Hall CB (2003). Respiratory syncytial virus: concerns and control. Pediatr Rev.

[7] Office of the Chief of Medical Officer of Health (2014). Nunavut Immunization Mannual. Palivizumab for Respiratory Syncytial Virus prevention. Iqaluit, Nunavut: Government of Nunavut.

[8] Homaira N, Rawlinson W, Snelling TL, Jaffe A (2014). Effectiveness of palivizumab in preventing RSV hospitalization in high risk children: a real-world perspective. Int J Pediatr.

[9] Figueras-Aloy J, Carbonell-Estrany X, Quero J; IRIS Study Group (2004). Case-control study of the risk factors linked to respiratory syncytial virus infection requiring hospitalization in premature infants born at a gestational age of 33-35 weeks in Spain. Pediatr Infect Dis J.

[10] Nishimura T, Suzue J, Kaji H (2009). Breastfeeding reduces the severity of respiratory syncytial virus infection among young infants: a multi-center prospective study. Pediatr Int.

[11] Holman RC, Shay DK, Curns AT, Lingappa JR, Anderson LJ (2003). Risk factors for bronchiolitis-associated deaths among infants in the United States. Pediatr Infect Dis J.

[12] Figueras-Aloy J, Carbonell-Estrany X, Quero-Jiménez J, Fernández-Colomer B, Guzmán-Cabañas J, Echaniz-Urcelay I, Doménech-Martínez E (2008). FLIP-2 Study: risk factors linked to respiratory syncytial virus infection requiring hospitalization in premature infants born in Spain at a gestational age of 32 to 35 weeks. Pediatr Infect Dis J.

[13] Simoes EA (2003). Environmental and demographic risk factors for respiratory syncytial virus lower respiratory tract disease. J Pediatr.

[14] Meissner HC (2004). The unresolved issue of risk factors for hospitalization of infants with respiratory syncytial virus infection born after 33-35 weeks gestation. Pediatr Infect Dis J.

[15] Law BJ, Langley JM, Allen U (2004). The pediatric investigators collaborative network on infections in Canada study of predictors of hospitalization for respiratory syncytial virus infection for infants born at 33 through 35 completed weeks of gestation. Pediatr Infect Dis J.

[16] Welliver RC (2003). Review of epidemiology and clinical risk factors for severe respiratory syncytial virus (RSV) infection. J Pediatr.

[17] Thomas W, Speer CP (2005). Bronchopulmonary dysplasia: epidemiology, pathogenesis and treatment. Monatsschrift Kinderheilkd.

[18] Camargo CA Jr, Ingham T, Wickens K (2011). Cord-blood 25-hydroxyvitamin D levels and risk of respiratory infection, wheezing, and asthma. Pediatrics.

[19] Belderbos ME, Houben ML, Wilbrink B (2011). Cord blood vitamin D deficiency is associated with respiratory syncytial virus bronchiolitis. Pediatrics.

[20] Henrickson KJ, Hoover S, Kehl KS, Hua W (2004). National disease burden of respiratory viruses detected in children by polymerase chain reaction. Pediatr Infect Dis J.

[21] Ruuskanen O, Ogra PL (1993). Respiratory syncytial virus. Curr Probl Pediatr.

[22] Sommer C, Resch B, Simões EA (2011). Risk factors for severe respiratory syncytial virus lower respiratory tract infection. Open Microbiol J.

